# Towards standardization guidelines for *in silico* approaches in personalized medicine

**DOI:** 10.1515/jib-2020-0006

**Published:** 2020-07-24

**Authors:** Søren Brunak, Catherine Bjerre Collin, Katharina Eva Ó Cathaoir, Martin Golebiewski, Marc Kirschner, Ingrid Kockum, Heike Moser, Dagmar Waltemath

**Affiliations:** University of Copenhagen, Copenhagen, Denmark; HITS gGmbH, Heidelberg, Germany; Forschungszentrum Jülich GmbH, Project Management Jülich, Jülich, Germany; Karolinska Institutet, Solna, Sweden; Medical Informatics Laboratory, Institute for Community Medicine, University Medicine Greifswald, Greifswald, Germany

**Keywords:** data integration, *in silico* modelling, personalized medicine, reproducibility, standards

## Abstract

Despite the ever-progressing technological advances in producing data in health and clinical research, the generation of new knowledge for medical benefits through advanced analytics still lags behind its full potential. Reasons for this obstacle are the inherent heterogeneity of data sources and the lack of broadly accepted standards. Further hurdles are associated with legal and ethical issues surrounding the use of personal/patient data across disciplines and borders. Consequently, there is a need for broadly applicable standards compliant with legal and ethical regulations that allow interpretation of heterogeneous health data through *in silico* methodologies to advance personalized medicine. To tackle these standardization challenges, the Horizon2020 Coordinating and Support Action EU-STANDS4PM initiated an EU-wide mapping process to evaluate strategies for data integration and data-driven *in silico* modelling approaches to develop standards, recommendations and guidelines for personalized medicine. A first step towards this goal is a broad stakeholder consultation process initiated by an EU-STANDS4PM workshop at the annual COMBINE meeting (*COMBINE 2019 workshop report in same issue*). This forum analysed the status quo of data and model standards and reflected on possibilities as well as challenges for cross-domain data integration to facilitate *in silico* modelling approaches for personalized medicine.

## 1 Introduction

A key strategic objective of EU-STANDS4PM is to engage with relevant stakeholders. Through specific awareness actions, including workshops, the project provides a forum to assess strategies for health data integration (such as genetic, expression, proteomics, demographic, clinical, and lifestyle exposures) as well as data-driven *in silico* modelling1
*In silico* modelling, in this context refers to mathematical and computational models of biological systems, such as molecular modelling, modelling of subcellular processes, individual-cell or cell-based models, tissue/organ level models, body systems level models (1. Wolkenhauer O, Auffray C, Brass O, Clairambault J, Deutsch A, Drasdo D, et al. Enabling multiscale modeling in systems medicine. *Genome Med*. 2014;6). [1] approaches. This proactive networking process is central to the major deliverable of EU-STANDS4PM, which is to jointly develop universal standards as well as guidelines and recommendations for *in silico* methodologies relevant for personalized medicine in a concerted action.

To initiate this process, EU-STANDS4PM consulted the COMBINE (Computational Modelling in Biology Network) community to address crucial requirements with respect to the development of data and model standards as well as data integration tasks in research and clinic, including ethical and legal aspects. These areas were discussed at the annual meeting of COMBINE in moderated round table workshops covering six central topics presented below and that conclude in a first set of recommendations to key actors.

## 2 Standards as drivers for reproducibility and data quality

As a major requirement for any scientific result reproducibility ensures high quality of investigations and vice versa high-quality investigations ensure reproducibility. Particularly, for personalized medicine, reproducibility of clinical research and development ensures patient safety. Even more than in classical modelling applications, revision-safe and reproducible documentation of studies is necessary when using modelling as a tool in clinical research settings. Standards may not be necessary to replicate a result, but they are indeed the drivers for reproducibility and they ensure higher quality of data, thus increasing trust in the scientific findings. Most standards in systems medicine are so-called *de facto standards*, meaning they are not legally binding. However, regulations for medical treatments are backed up by law and documentation, for example, in a hospital. They must be revision safe. This gap needs to be bridged and hence existing standards must be moved to the level of *de jure standards*, especially for cases that require revision safety (i.e. ensuring that all data and software code is being archived in a verifiable and transparent manner that allows to obtain the original, unchanged data or software code at later points in time).

To move systems medical standards to the level of *de jure* standards, it will be necessary to have them approved by formal authorities like the International Organization for Standardization (ISO). These organizations offer a critical and thorough assessment of all elements of a standard before release. Particularly, for the implementation of virtual studies in the clinic – that is the investigation in a biomedical system by means of modelling and simulation [2] – it is indispensable to ensure traceability of all analysis steps, boundary conditions, and assumptions through proper standardization. A first step into formalizing systems medicine standards has already been taken with the work of EU-STANDS4PM in different ISO and German Institute for Standardization (DIN) committees (see section “Community and Formal Standards” below).

Today virtual studies already predict disease progression [3], [4], support decision making [5], enable cross-validation of possible treatment outcomes [6] and are used for educational purposes [7]. They typically consist of (i) one or more models, (ii) one or more simulations, and (iii) a collection of results. These three ingredients need to be well-documented and each component must be tested for correctness. Reproducibility then requires standard formats to represent the data, detailed descriptions following the Good Scientific Practices described in Minimum Information Guidelines, and semantic annotations [8], [9]. The computational biology community has already developed standards for all parts of a typical virtual study and the authors are convinced that these well-established COMBINE standards [9] shall be thoroughly evaluated for use in predictive *in silico* models in personalized medicine. Equally important is the correctness of the software code to run a computational model. In addition, certification procedures, usually complex and time consuming, will be necessary for any model to be run in a clinical setting. One step into this direction, are efforts by regulatory authorities, such as the US Food and Drug Administration (FDA) and industry, to advance the use of computational modelling as medical advices, including the development of the respective standards [10], [11].

Finally, standards and standard operating procedures are necessary for the definition and execution of software pipelines; this applies in particular to complex simulations. Software pipelines, for example built-in workflow systems like Galaxy [12], can in themselves be considered software products, requiring similar procedures as for the model code and the simulation software. Furthermore, the COMBINE Archive format is the one development to allow for the exchange of multi-file experiments [13] and first applications of eNotebooks and docker have proven successful [14], [15], [16].

In summary, standards are needed to encode data, model(s), simulation, and results – but furthermore, standards need to be evaluated for method development and protocols, for documents in general and for meta-data encoding. Although many community projects and their efforts in developing standards are of considerable value for personalized medicine, satisfying the strict requirements of clinical evaluation of security and reporting guidelines are remaining and high hurdles.

## 3 Community and formal standards

Standards provide a specific set of rules, guidelines and characteristics to ensure that products, processes and services (including data and models) fit the individual purpose of the users and consumers in the best possible way. As such standards record in a comprehensible manner what information users can expect in a data set and, at the same time, specify which rules, requirements or conditions should or shall be followed. In the context of personal/patient-derived data in a clinical setting, standards are needed to ensure data security, quality and availability for any data-driven application. However, especially in the life sciences and in clinical research, there are still many challenges associated with defining what a standard really is and there are basically two independent worlds developing formal and community standards. In the following section, we will analyse the major differences, and briefly, discuss the challenges associated with bridging these two systems.

### 3.1 Formal standards

All formal standards are created by official international standardization bodies (Table 1). Their development is based on the consensus principle, in a defined procedure with the participation of all interested stakeholders. Given the consensus mechanisms of ISO, the development time for ISO standards is typically 3–5 years. During the development of a new formal standard, existing regulations will be considered as much as possible. A formal standard, once completed and released, is internationally respected and recognized as state of the art – also from a legal perspective. ISO standards are persuasive and hence provide users with a high degree of planning security – their definition is sustainable for many years by means of regular status quo assessments (every 5 years for an ISO standard) and subsequent adjustment procedures. Commercial standards are taken into account as far as they are known. These standards vary widely and depend on who created them (e.g. a small research group or a large company) and what the intention was when they were created (e.g. to provide interoperable data for customers or to secure own market advantages). Accordingly, the specifications and requirements of such standards are more or less easily transformed into formal standards. In the case of securing the market of a group or company, it is often only possible to establish formal standards as interface or mapping standards to commercial standards, and this only whether the customers strongly insist on it. Nevertheless, all groups developing formal standards try to take commercial standards into account as far as they are known. However, it will never be possible to know all commercial standards. Only the portfolio of formal standards shall be free of any contradictions.

**Table 1:** Examples of internationally accepted standard bodies.

Internationally accepted standard bodies	
Level	Standard body
International	International Organization for Standardization (ISO) International Electrotechnical Commission (IEC)
European	European Committee for Standardization (CEN) European Committee for Electrotechnical Standardization (CENELEC) European Telecommunications Standards Institute (ETSI)
National^a^	Association Française de Normalization (AFNOR) British Standards Institution (BSI) German Institute for Standardization (DIN) Danish Standards (DS) Royal Netherlands Standardization Institute (NEN) Swedish Institute for Standards (SIS)

a A comprehensive list of e.g. ISO national members can be found under: www.iso.org/members.html.

### 3.2 Community standards

In comparison to their formal counterparts, community standards usually reflect the results of a specific user group and are created by individual enterprises or communities such as COMBINE [17]. As such, community standards typically cover a broad variety of different topics from basic business models to data sharing (e.g. Findable, Accessible, Interoperable, Reproducible – FAIR-guiding principles) [18] or Good Epidemiological Practice [19], [20]. There is no prescribed process for creating, agreeing, and consensus-building – but also no time frame. Therefore, community standards are usually available within a relatively short time.

The use of both, formal and community standards, is on voluntary base. However, for a community standard, there is no obligation to adhere 100% to the regulations which means that minor adjustments are possible. Own special requests for individual modifications are therefore comparably simple to realize – an approach not possible for modifying a formal standard.

### 3.3 Challenges and hurdles

In the case of community standards, it is not always clear, whether they have been established on a broad scientific basis or not. Therefore, an evaluation of these standards is often necessary before they can be applied. Careful verification determines whether a standard fits a new application and if so, whether the efficacy and benefit is also given. However, participation in a formal standardization project is lengthy, requires many resources and often exceeds the duration of research projects as well as the time for which scientific personnel is funded. Even if a formal standardization process is relevant for the sustainability of a research project, this is often not taken into account when applying for funds for research projects. In such as case, there is a high chance that funds cease before this process has ended.

## 4 Pitfalls in developing and harmonizing standards

The development of standards in the life sciences, especially for personalized medicine, is a challenging task. First of all, a substantial amount of time and effort is needed to define a standard for a certain purpose and field, as it should cover not only one, but many potential use cases. Such an investment of time and resources is demanding, if possible at all, for researchers who deal with standardization as a side job. On the other hand, and if the necessary resources are available, chances are that the development of a standard is driven distinctively by the researchers’ genuine scientific work. This can lead to a biased standard tailored towards a specific topic, process or product. Thus, not necessarily the best – or most appropriate for the scientific community at large – standardization concept wins, but the one with the most supporting resources. This situation provides a competitive advantage for those stakeholders involved in the development of the winning concept. Sometimes there are even competing standards, as for example, in the field of life science microscopy imaging with a whole range of different, mostly proprietary standards [21]. A more recent example is the development of standards for genome compression [22], [23]. In the most extreme cases, such a competition can lead to an overrun of existing and well-established standards by newly developed ones.

A lack of maintainability adds to the difficult situation. Standards development (and their implementation in software tools) requires long-term available resources that are often not present given the current system of research funding (see above). Standards need to be maintained in a sustainable manner, also beyond the first release of a standard; otherwise, they become outdated. The additional problem of competing standards is especially crucial for the field of personalized medicine. Here, the obligation of long-term storage of patient data (and data derived from the primary patient data) makes sustainability a prerequisite.

The long development time for most standards (up to 5 years through the ISO route) may even lead to the preposterous situation that a standard can already be outdated once released. On the other hand, especially in evolving and modern scientific fields such as personalized medicine, a standard developed too early, based on technological methods that are not mature enough, might not cover all major aspects. Thus, the timing of standardization is absolutely crucial.

A central problem when developing standards is acceptance by the whole research community. Developed standards become useless if not known to the community or not properly adopted by software tools, researchers and/or clinicians. Thus, it is crucial for the acceptance that a standard is drafted by a representative part of the community of the corresponding domain, and not by single individuals or institutions that would like to promote their own workflows or tools. That ensures that a standard reflects the best practice in the corresponding domain. However, even when a standard is developed by a representative part of the community that is dedicated to put efforts into standardization, it still might be unknown to the majority of the scientific community. Consequently, standards have to be promoted in the communities, and they have to be developed close to existing workflows and data structures models to enable simple implementation procedures, ensuring a wide-spread adoption of the standard. For the same reasons, it is crucial that regulatory and governance bodies releasing and monitoring regulatory standards work closely with the scientific communities when establishing such official and mandatory standards. Moreover, over-standardization should be avoided to not hinder the dynamic development in a scientific field and hence the loss of flexibility therein. This is of crucial importance in personalized medicine, as here the field is quickly developing while, at the same time, highly regulated by authorities.

## 5 Standards relevant for personalized medicine

**Figure 1:**
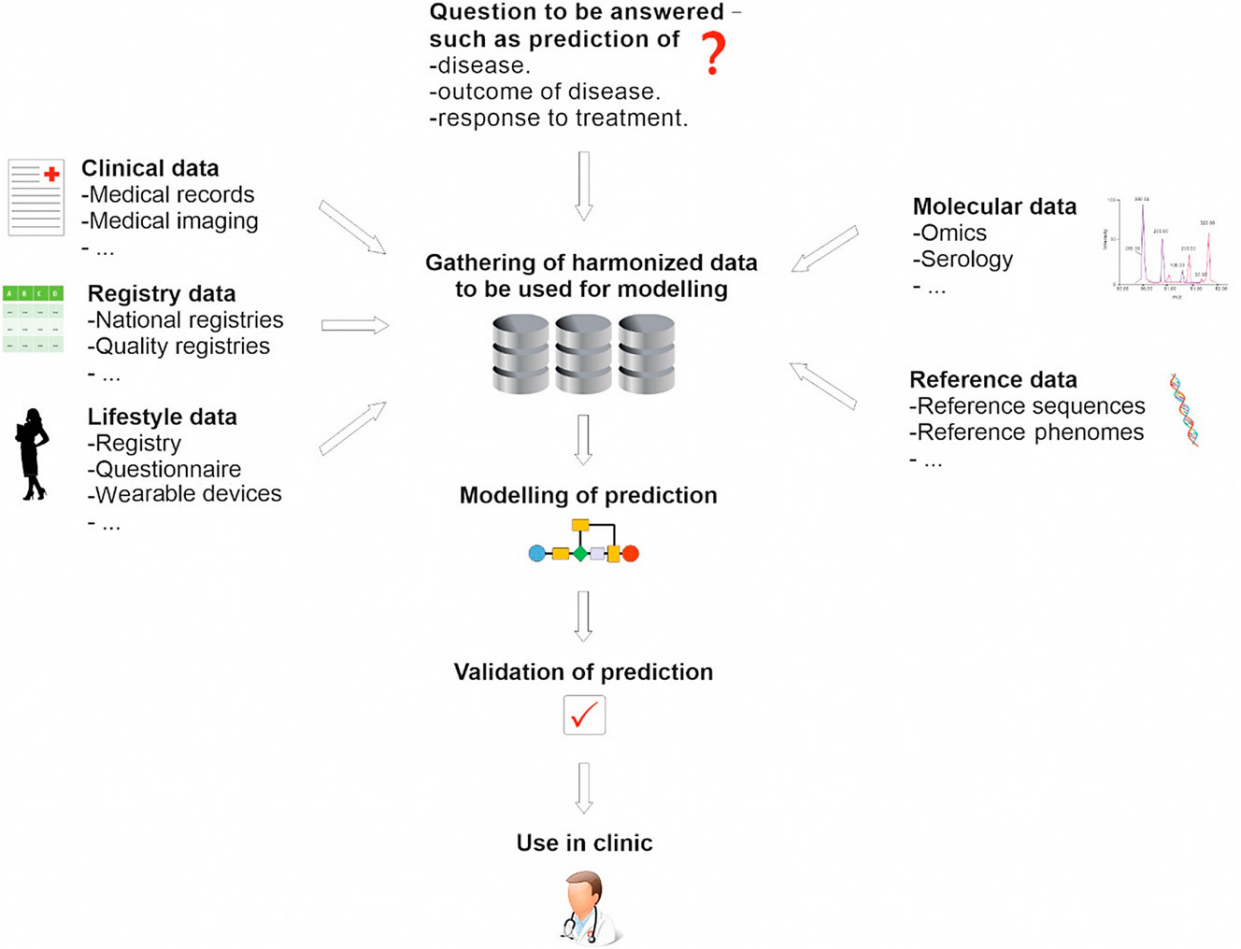
A typical workflow for personalized medicine. A personalized medicine approach to improve patient health typically starts with identifying which aspect of health that is to be addressed and modelled, for example prediction of disease, prediction of severity of a particular disease or prediction of response to treatment. The next step is typically identification of relevant data sources, which can be of many different types, as illustrated. These data often have to be harmonized, a task that is made easier whether common data standards have been used. Once this has been completed, modelling to predict the clinically relevant state takes place. Usually the models then have to be validated in an independent setting. Once that has been completed, the models can be used in a clinical environment to help improve patient health. Picture source (licence free): www.pixabay.com.

The future development of personalized medicine is dependent on an exchange of data from different sources. Patients will benefit from computational models that predict treatment outcomes. For example, models of a specific molecular characterization can be tested based on broad exchange of data from different clinical studies. This will allow comprehensive comparison of treatment outcomes under different settings and predictions for an optimal individual and personalized therapy. Figure 1 illustrates a typical workflow followed in personalized medicine starting with the clinical question, followed by identification, access, and harmonization of relevant data, the development of data model(s), the model validation and finally application in a clinical setting.

A successful interchange of data, whether sensitive/personal or not, is highly dependent on the standardization of data sources and encoding of models. Different types of data (e.g. genetic, expression, proteomics, demographic, clinical, and lifestyle exposures) can be of relevance for personalized medicine. Data standards already exist for many types of data [24] and are widely used (Table 2); while for other types of data, there are very few established standards. In the following section, we will briefly discuss the latter ones and highlight specific challenges.

**Table 2:** Common standards relevant for personalized medicine and *in silico* approaches.

Common standards relevant for personalized medicine	
*DNA, RNA, protein sequence formats*	
FASTA	Widely used for representing nucleotide sequences or amino acid, developed for use in the FASTA programme [44], [45].
Sequence alignment/map (SAM) and BAM format	Capture of sequences that have been aligned to a reference genome. BAM is in a binary more condensed version while SAM has the same information in a series of tab delimited ASCII columns [46].
CRAM	A compressed columnar file format also used for storing biological sequences mapped to a reference sequence, it has been developed to improve compression and hence save on storage costs [47].
General feature format (GFF)	Stores DNA, RNA or protein genetic sequence data [4]. It stores the whole sequence for the relevant feature.
Variant call format (VCF)	A text format file storing the same data but only contains the sites which differ from a given reference and hence is more space efficient than GFF [48]. Originally designed to be used for SNPs and INDELs but can also be used for structural variation.
Binary variant call format (BCF)	A binary version of VCF and therefore is more space efficient, the relationship between BCF and VCF being similar to that between BAM and SAM.
*Mass spectrometry*	
mzML	Stores the spectra and chromatograms from mass spectrometry in and eXtensible Markup Language (XML) format. Now a well-tested open-source format for mass spectrometer output files that is widely used [49].
mzTab	A more easily accessible format which could be used with R or Microsoft Excel tools in the field of proteomics and metabolomics. mzTab files can contain protein, peptide, and small molecule identifications. In addition, experimental meta-data and basic quantitative information [50].
*Medical imaging, Digital Imaging and Communications in Medicine*	
Digital Imaging and Communications in Medicine (DICOM)	Dominating standard used in medical radiology for handling, storage, printing and exchanges of images and related information. Specifies the file format and communication protocol for handling these files. Captures pixel data making up the image and how the image was generated (e.g. used machine and protocol, information regarding what patient the image is capturing. Living standard regularly maintained and modified [51].
The European Data Format (EDF)	A standard to archive, share, and analyse data from medical time series [52]
*Semantic integrations*	
Human Phenome Ontology (HPO)	Developed by the Monarch Initiative a consortium, carrying out semantic integration of genes, variants, genotypes, phenotypes, and diseases in a variety of species allowing powerful searches based on ontology. HPO is a standardized vocabulary of phenotypic abnormalities associated with disease. Standard terminology for clinical “deep phenotyping” in humans, providing detailed descriptions of clinical abnormalities and computable disease definitions [53]. The primary labels use medical terminology used by clinicians and researchers. These are complemented with laypersons synonyms. HPO is one of the projects in the Global Alliance for Genomics and Health (GA4GH) seeking to enable responsible genomic data sharing within a human rights framework [54].
*Tools and analysis pipelines*	
CellML	A standard based on XML markup language [55] used for storing and exchanging computer-based mathematical models allowing sharing of models even when different modelling tools are used [56].
The Systems Biology Markup Language (SBML)	A standard model interchange language that permits exchange of models between different software tools [57].
The Synthetic Biology Open Language (SBOL)	A standard to support specifications and exchange of biological design information [58].
Simulation Experiment Description Markup Language (SED-ML)	Developed to capture the Minimum Information about a simulation experiment (MIASE), the minimal set of information needed to allow reproduction of simulation experiments. SED-ML encodes this information in an XML-based computer-readable exchange format it was developed as community project [9], [59].
NeuroML	XML-based standardized model description language to describe mathematical models of neurons and complex neuronal networks [60].
PBPK/PD	Physiologically based Pharmacokinetic/Pharmacodynamic models allow a mechanistic representation of drugs in biological systems [61].

Examples of common standards that have been developed by specific user communities and different stakeholders. Their use has been enhanced as they have been coupled to tools which have spread in the respective field of research.

### 5.1 Lack of common data schemes

Processing pipelines for the analysis of raw data are rarely provided by researchers and are often not required during the publication process. However, to make underlying analysis of data as transparent as possible, such pipelines should be implemented with the actual raw data material and easily made available at the time of publication. Analysis pipelines such as the common workflow language (CWL) [25] already are established standards and provide a suitable tool, also in combination with the tool registry service (TRS) [26] for describing complex analysis workflows in different hard- and software environments.

### 5.2 Lack of data visibility, transparency of usage and duplications

In addition to the above discussed pipelines, many research publications lack sufficient traceable information on how scientific results were obtained or how specific conclusions were drawn, and currently, there are also no standardized mechanisms of how to request raw data to reanalyse findings [27]. Consequently, due to a lack of quality control mechanisms, reproducibility issues arise [28] and reuse of underlying data remains challenging or even impossible [29].

In the case of genomic and health-related data, many community-based standards already have been developed that are able to provide a harmonized data governance structure [24]. These standards, such as ELIXIR Beacon and MatchMaker [30], can help to identify individuals carrying specific polymorphisms and aid in making data more visible; sensitive data can be queried using meta data for certain characteristics prior to going through controlled access approval and transfer of data to the analyst [31]. In fact, transfer of data may not be needed at all if analysis is done in a cloud computing environment or if the analysis pipeline is transferred to the data instead of transferring the data to the analyst [32]. A related issue that requires further discussions is how to achieve higher transparency of data use, since there is a demand to record what a certain dataset is re-used for [33] – just as it should obviously be stated where data comes from, if re-used in publications [34], [35]. We hypothesize that the recent rise of data-oriented journals will push developments in these directions.

### 5.3 Lack of standards for life-style data

There seems to be a general lack of standards for life-style data, both when it comes in the form of data from wearable devices and when it comes from more traditional data sources such as questionnaires or medical records.

### 5.4 Lack of data harmonization

Medical language can be diverse, represented by different data types from document-oriented text mining results to data-oriented medical records. Combining these types of data is a highly challenging task – even more for cross-border data exchange and when stored in different national languages. There is a strong need for harmonization of capture of clinical data, such as input data for computational models. Systems such as openEHR [27] are able to support harmonization efforts by being an open standard specification describing the management, storage, retrieval and exchange of health data in electronic health records. The idea being that health data for a person is stored in a single vendor-independent person centred electronic health record.

## 6 Integration of clinical and research data

The integration of clinical and research data, while essential, is not trivial. Challenges include establishing semantically consistent disease annotations and medical vocabulary, handling different types of patient populations and overcoming highly diverse registration procedures of measurements and interventions. Finally, different legal and ethical rules apply to clinical and research data in European countries (and beyond). While some of the problems arise due to differences inherent in the data capture setting (patient populations are inherently different from research populations, research projects can allot more resources to information gathering that does not directly affect patient treatment), most of the challenges are similar to any data integration effort between different medical systems, be they clinical or research based.

For the purposes of data integration, the distinction between research and clinical data is narrowing, as systematized data collection and knowledge generation are becoming a characteristic not only of research but also of clinical practice [36], [37].

As clinical data become more systematized, digitized, and linkable, researchers are confronted with well-known data integration problems when working with clinical and research data. One difference in integrating these different data may be that clinical care data are very complex and less harmonized than most project/research-generated data, since the data sets are larger and patients are not stratified, thus heterogeneous, and often multi-morbid. Data can be integrated in different ways, and with different levels of identifiability.

### 6.1 Individual level integration

Research and clinical data can be linked at the personal identification-level so that research-generated and clinical care data become a combined pool of knowledge about a given individual. As the different data sources contribute different variables, linked to a given individual, this type of research/clinical data integration can be done without mapping data to uniform ontologies. Missing data and contradictions can be handled through analysis of the data source and data creation route, and extremely valuable knowledge is generated throughout projects.

### 6.2 Integration of variables

Data can be combined at the variable level, for example diagnoses may be integrated from national records using International Statistical Classification of Diseases and Related Health Problems (ICD)-codes, projects using project-based definitions of disease, and questionnaires using self-identification by patients. Blood sugar values may be combined from national patient journal lab records, project lab data with different equipment and normal values, and patient home self-measurements. This obviously requires large mapping efforts to standardized mapping concepts and produces unreliable data integration and results.

Solutions used in the integration of large-scale, transnational project data from multiple clinical centres [38] could be applied to address the above challenges, for example calibration of lab values to handle inter-lab variations and techniques to impute missing values.

Clinical and research data can contribute to joint results by training an algorithm sequentially on the data sets without combining them. Validating results derived from clinical data, using research data (or the other way around) is also a way of avoiding having to combine data sets governed by different ethical, legal, and security constraints. However, the data sets still have to be standardized and interoperable to return useful results. Therefore, data generated both by research projects and clinical care should be designed for interoperability.

## 7 Using patient-derived data for personalized medicine: legal and ethical aspects

Legal and ethical governance of personalized medicine at European level is composed of a patchwork of international, regional, and national laws, as well as non-binding recommendations, that seek to protect patients from breaches of their privacy and confidentiality, and from discrimination on the basis of health data [39]. This spans international bioethics treaties from the Council of Europe (CoE), namely the Biomedicine Convention [40], and non-binding recommendations on bioethics drafted by CoE and United Nations Educational, Scientific, and Cultural Organization (UNESCO) [41]. At European (EU) level, the European Union General Data Protection Regulation (GDPR) entered into force in 2018 and has important implications for the processing of personal data in treatment and research contexts [42]. However, this legal and ethical framework is subject to several challenges. Bioethical and legal norms were established post World War II with research on human subjects in mind, not computational models, meaning that regulations often do not fit the big data landscape. Furthermore, international treaties, including the GDPR [43], leave significant discretion to national legislatures, resulting in varied implementation among member states and potential legal uncertainty. In the case of CoE norms, there are limited means of enforcement, in comparison with GDPR where breaches can result in significant fines.

Regarding the development of *in silico* models, there are several challenges. Firstly, while the GDPR has direct effect in all EU member states, it also provides for national variations. Crucially for researchers, Article 89 GDPR allows member states to enact derogations from the rights referred to in Articles 15, 16, 18, and 21 when processing personal data for scientific research purposes. This can lead to national variances that have implications for cross-border research collaboration. In other words, despite the aim of harmonization, national data protection legislation continues to differ among member states and may place varying requirements on researchers who inevitably conduct research across borders.

*In silico* modelling also raises concerns regarding the type of information that must be supplied to research subjects to meet GDPR consent requirements. This can be a challenge for *in silico* models where the research hypothesis is unclear. Another concern surrounds the principle of data minimization (data should be limited to what is necessary) as enshrined by Article 5(1)(c) GDPR and the level of anonymization required for data to avoid falling under GDPR.

From an ethical perspective, willingness to donate data, specifically whether the public has adequate knowledge of the possible implications, is of concern. The familial implications of donating one’s genetic data for research/other purposes also require study.

## 8 Conclusions

A key output of the discussed workshop topics was a summary of challenges associated with the implementation of data-driven *in silico* methodologies in personalized medicine and clinical practice. In this section, we highlight these challenges and provide a set of recommendations directed towards different key actors.

### 8.1 Funders, including the EU-commission

Key requirements of any grant funding for personalized medicine projects should be that: (i) Grant recipients make algorithms and pre-processed project data available to the community and (ii) algorithms are accompanied by documentation and follow approved standards. Standardization efforts shall also be fully fundable to ensure that appropriate and sufficient resources are made available to the scientific communities for developing standards that the researchers then could apply consistently to their workflows. This ensures establishment of standards that reflect best practice in their domain. Data processing, documentation, and subsequent sharing thereby become integral, obligatory deliverables of funded projects, included in the budget and planning. Data sharing and documentation thereby become less onerous than currently, where they are un-funded and altruistic.

### 8.2 Healthcare providers purchasing and developing electronic healthcare systems

State organizations purchasing healthcare systems should make data harvesting a criterion for system developers and providers. Many providers regard both the data produced and the algorithms involved as proprietary and create closed systems where analysis of data proceeds internally with key limitations in how data analysis can be performed. This is, for example, the case with some providers of Electronic Patient Record systems, where the business model seems to work against open systems. Instead, we suggest that tools should be shared even across countries, healthcare providers, and with academic or industrial stakeholders involved in health data science. The negotiation power necessary to enforce harvestability of these data might arise only as a consequence of legislation making it compulsory.

### 8.3 Journals

A requirement of publication should be processed data deposition in recommended, preferably open data repositories. Where the nature of the data is such that deposition is not legal/ethical, a description of the data should be catalogued in such a repository. Restricted access models will also in many cases be needed and desirable.

### 8.4 Research groups

Documentation and data sharing tasks should be included in the preparation of grant applications for projects in the form of a data management plan. Once the project is initiated, documentation should be prioritized when pre-processing data to make it possible for others to re-use processed data. Algorithms should follow available standards unless there are clear reasons why not to use such existing standards. The advantage of being cited for re-use of pre-processed data and algorithms should be a focus point. Transparency and compliance with standards for algorithms and data should be a key quality parameter when assessing both one’s own work, and work received for peer review.

### 8.5 National and regional health data providers

Options for sharing of pre-processed data originally provided by these actors should be facilitated. For example, the Health Data Authorities could provide a repository for pre-processed data and scripts, stipulating that the researchers having done the pre-processing must be credited in work building upon it. Too often, users are handed poorly annotated data requiring cleaning in the same way leading to substantial duplication of effort. Guidelines for returning clean and value-added data to data providers should be encouraged.

### 8.6 Policy makers

To ensure best adaptation and acceptance of mandatory standards, regulatory and governance bodies, as well as other policy makers releasing and monitoring such standards should work closely with the scientific communities when establishing official standards. The need for greater clarity regarding the scope of legal standards related to personalized medicine is clear. Treaties and recommendations should be reconsidered in light of big data-driven healthcare. Yet, even newer legislation, namely the GDPR, is open to interpretation and national deviation, which can leave researchers and individuals unclear regarding processing of personal data. This should be addressed through legal guidance from, for example, the European Court of Justice. Furthermore, there is a need for greater transparency within the healthcare system regarding use of data for research, including informed opportunities to opt out of secondary use and information on data ownership. Governments should ensure that individuals are adequately protected from misuse of their data, including through proportionate fines. Although scientific research is vital, the individual’s rights continue to weigh higher in international bio law.

## EU-STANDS4PM consortium

Rolf Apweiler^1^, Stephan Beck^2^, Catherine Bjerre Collin^3^, Niklas Blomberg^1^, Søren Brunak^3^, Tom Gebhardt^4^ Eugenijus Gefenas^4^, Martin Golebiewski^6^, Kalle Günther^7^, Mette Hartlev^3^, Vincent Jaddoe^8^, Marc Kirschner^9^, Ingrid Kockum^10^, Sylvia Krobitsch^9^, Lars Küpfer^11^, Stamatina Liosi^2^, Vilma Lukaseviciene^5^, Ali Manouchehrinia^10^, Arshiya Merchant^1^, Neha Mishra^12^, Heike Moser^13^, Miranda Mourby^14^, Wolfgang Müller^5^, Flora Musuamba Tshinanu^15^, Katharina Eva Ó Cathaoir^3^, Uwe Oelmüller^7^, Tito Poli^16^, Philip Rosenstiel^12^, Dagmar Waltemath^17^, Olaf Wolkenhauer^4^, Amonida Zadissa^1^

^1^ European Bioinformatics Institute (EBI-ELIXIR), United Kingdom

^2^ University College London, United Kingdom

^3^ University of Copenhagen, Denmark

^4^University of Rostock, Germany

^5^ Vilnius University, Lithuania

^6^ HITS gGmbH, Germany

^7^ Qiagen GmbH, Germany

^8^ Erasmus University Rotterdam, The Netherlands

^9^ Forschungszentrum Jülich GmbH, Project Management Jülich, Germany

^10^ Karolinska Institutet, Sweden

^11^ Bayer AG, Germany

^12^ University of Kiel, Germany

^13^ German Institute for Standardization, Germany

^14^ Univeristy of Oxford, United Kingdom

^15^ Federal Agency for Medicines and Health Products, Belgium

^16^ University of Parma, Italy

^17^ Medical Informatics Laboratory, Institute for Community Medicine, University Medicine Greifswald, Germany
